# Developing recommendations to improve the quality of diabetes care in Ireland: a policy analysis

**DOI:** 10.1186/1478-4505-12-53

**Published:** 2014-09-18

**Authors:** Sheena M Mc Hugh, Ivan J Perry, Colin Bradley, Ruairí Brugha

**Affiliations:** Department of Epidemiology & Public Health, University College Cork, 4th Floor, Western Gateway Building, Western Rd., Cork, Ireland; Department of General Practice, University College Cork, Room 2.41 Western Gateway Building, Western Rd, Cork, Ireland; Department of Epidemiology & Public Health Medicine, Royal College of Surgeons Ireland, Beaux Lane House, Mercer Street Lower, Dublin 2, Ireland

**Keywords:** Health care reform, Policy analysis, Quality of care, Type 2 diabetes mellitus

## Abstract

**Background:**

In 2006, the Health Service Executive (HSE) in Ireland established an Expert Advisory Group (EAG) for Diabetes, to act as its main source of operational policy and strategic advice for this chronic condition. The process was heralded as the starting point for the development of formal chronic disease management programmes. Although recommendations were published in 2008, implementation did not proceed as expected. Our aim was to examine the development of recommendations by the EAG as an instrumental case study of the policy formulation process, in the context of a health system undergoing organisational and financial upheaval.

**Methods:**

This study uses Kingdon’s Multiple Streams Theory to examine the evolution of the EAG recommendations. Semi-structured interviews were conducted with a purposive sample of 15 stakeholders from the advisory group. Interview data were supplemented with documentary analysis of published and unpublished documents. Thematic analysis was guided by the propositions of the Kingdon model.

**Results:**

In the problem stream, the prioritisation of diabetes within the policy arena was a gradual process resulting from an accumulation of evidence, international comparison, and experience. The policy stream was bolstered by group consensus rather than complete agreement on the best way to manage the condition. The EAG assumed the politics stream was also on course to converge with the other streams, as the group was established by the HSE, which had the remit for policy implementation. However, the politics stream did not converge due to waning support from health service management and changes to the organisational structure and financial capacity of the health system. These changes trumped the EAG process and the policy window remained closed, stalling implementation.

**Conclusions:**

Our results reflect the dynamic nature of the policy process and the importance of timing. The results highlight the limits of rational policy making in the face of organisational and fiscal upheaval. Diabetes care is coming on to the agenda again in Ireland under the National Clinical Care Programme. This may represent the opening of a new policy window for diabetes services, the challenge will be maintaining momentum and interest in the absence of dedicated resources.

**Electronic supplementary material:**

The online version of this article (doi:10.1186/1478-4505-12-53) contains supplementary material, which is available to authorized users.

## Background

A government’s priorities are said to be reflected in the policies which have been established and implemented [1]. In Ireland, diabetes has floated in and out of the policy spotlight for a number of years, struggling to make it onto and stay on the agenda for action. While there are numerous recommendations and reports, there is no government-led national strategy document dedicated solely to the management of diabetes, akin to those for other conditions [2] or in other European countries [1]. Having a strategy to guide service development provides a foundation for the integration and continuity of chronic illness care [3].

Diabetes is an increasingly common chronic condition with the worldwide prevalence expected to reach over 4% of the population by 2030 [4]. As a result of severe and long-term complications, diabetes places a huge burden of care and cost on the individual, health care providers, and the health system. People with diabetes are more likely to be admitted to hospital than people without diabetes, and multiple hospitalisations are common [5]. In 2010, global health expenditure on diabetes was projected to total at least $376 billion United States Dollars (USD), rising to $490 billion USD by 2030 [6].

Over two decades ago, the St Vincent Declaration called for formal recognition of the problem of diabetes and the deployment of resources to tackle the condition, including the development of plans for its long-term management [7]. At that time, the Irish government was urged to form a dedicated policy planning group, given the disorganised nature of diabetes care outside the major cities and lack of service planning [8]. Most countries have long since shifted the focus of diabetes care from acute episodic care to regular structured management with increasing primary care involvement [9, 10]. In Ireland, diabetes services are characterised by substantial variation with multiple local arrangements in place including traditional hospital-based management, shared care between General Practitioners and hospitals, and primary care-led management. In many areas there is deficient access to allied health services to support multidisciplinary team-based management [11, 12]. While there are a number of special interest groups led by health care professionals, which have driven improvements in care at a local level [13–15], at the time of this study there was no national strategy for the development of diabetes services.

The lack of progress towards a national strategy was noted at European level in 2005 [1] and again in 2008 [16]. In 2005, 11 of 25 countries in the Europe Union had a national framework or plan for diabetes, rising to 13 of 27 member states by 2008. The 2008 report from the International Diabetes Federation European Region and the Federation of European Nurses in Diabetes referred to the anticipated outcome of the Expert Advisory Group (EAG) for Diabetes in Ireland, a “*strategic review of the provision of diabetes treatment and services*” (p. 46) [16]. The EAG was established by the Health Service Executive (HSE) in 2006 to act as the source of “*operational policy, strategy and quality standards*” for diabetes care (p. 1) [17]. The Diabetes EAG was intended to have positive effects beyond diabetes as the process was heralded as the starting point for the development of formal chronic disease management programmes in Ireland [18].

**Figure 1:**
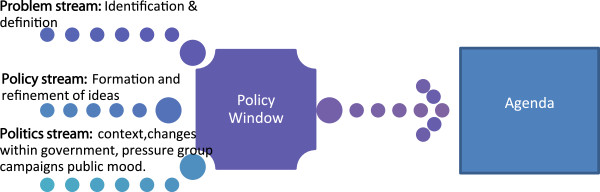
**Diagrammatical representation of the Multiple Streams Framework based on Kingdon [** [21] **].**

The aim of this study was to examine the development of recommendations by the EAG as an instrumental case study of the policy process. Although the EAG proposals were approved by the HSE in 2008 and a number of best practice guidelines and protocols were developed [19, 20], implementation of proposed changes did not follow as expected. Hence, this is a study of policy-making in the context of a health system undergoing major organisational and structural change, providing insight into the challenges and limits to rational policy making in the face of radical reform. We wanted to explore how the recommendations evolved in this context and why it happened in that way. We used the Multiple Streams Theory [21] to understand the evolution of the EAG recommendations. According to this framework, there are three largely separate streams running through the policy system: problems, policies, and politics. At a critical point known as the ‘policy window’ the three streams are coupled by policy entrepreneurs, moving an issue onto the agenda (Figure 1).

The problem stream is highlighted by indicators, a focusing event, or feedback on the success of current policy programs. Problem definition can result from people imposing their values or standards on a situation or through comparison within or between countries. The policy stream consists of ideas which tend to be a recombination of existing or familiar proposals that have been debated and revised over time. To survive, an idea must meet certain criteria: technical feasibility, value acceptability (proposals compatible with values of the policy community), and anticipation of future constraints (the chance of proposals being politically, publically, and financially acceptable). The politics stream refers to the broader political context within which policy is made, including changes in public mood and within government agencies. The agenda may be influenced by turnover (people change) or changes in jurisdiction (boundaries of responsibility change). The chances of an issue making it onto the decision agenda are increased by the coupling of all three streams; when the problem is recognised, feasible solutions are available and there is sufficient political will to support change [21].

## Methods

### Setting

The study was conducted in the Republic of Ireland. The HSE has been responsible for the provision of public healthcare services since 2005. The Department of Health is responsible for strategic planning in the health system including the formulation and evaluation of policy. Ireland was one of the countries worst affected by the recent global economic crisis, entering an economic recession in 2008 [22]. This had a significant impact on health care spending and the provision of services [23]. In 2009, a moratorium on recruitment in the health sector was introduced. Staff levels in the public health service have declined by 10% since 2007. The financing of the HSE fell by 22% between 2009 and 2013 [24]. As well as the introduction of cost-saving measures, there are ongoing organisational changes, including the establishment of a Quality and Clinical Care Directorate in 2010, plans to disband the HSE announced in 2012, and the phased introduction of universal health care purported to begin in 2014.

### Design

We used a qualitative instrumental case study design based on the classification of case studies by size and intent of analysis [25, 26]. In terms of size, this was a single case study design with defined boundaries in terms of timescale (lifecycle of the EAG from establishment in 2006 until the final meeting in July 2010) and participants (members of the EAG). In terms of the intent of analysis, this was an instrumental case study analysed to illustrate a type of policy formulation process commonly used in health policy to address major population-based programmatic policy decisions: involving expert groups in the evaluation and synthesis of evidence and the formulation of recommendations [27]. A case study investigates a contemporary phenomenon within a real-life context. Therefore, the subject of this study was the development of recommendations by the EAG within the context of the wider health and social system in Ireland. Ethical approval was granted by the Clinical Research Ethics Committee of the Cork Teaching Hospitals.

### Participants

A purposive sampling method was used to select professionally and geographically diverse interviewees based on their involvement in the EAG. The EAG included experts who volunteered from general practice, endocrinology, paediatrics, nursing, pharmacy, dietetics, biochemistry, and public health, and also representatives from the main patient association, the Department of Health and Children, and senior levels of the HSE. Interviewees were invited by letter or email, explaining the objective of the study. In total, 15 of the 22 members of the group were interviewed (68% response rate). Reasons for non-participation included relocation and workload, while other members did not respond to correspondence. Upon circulation of the transcripts, one participant withdrew leaving a final sample of 14 interviews for analysis.

### Data collection

Data were collected between August 2010 and January 2011. A detailed case description was built through in-depth data collection using multiple sources. The primary method was semi-structured key informant interviews conducted by one author (SMH). Interviews took place between November 2010 and January 2011. A topic guide was initially formulated using the policy analysis triangle focusing on the policy content, context, actors, and policy processes [28]. The sequence and content of the topic guide was organised around the pathway for developing evidence-informed policy [29] (see Additional file 1 for a copy of the topic guide). At the end of each interview, participants were asked if they wished to add anything to the discussion or raise any issue that had not been covered. Participants were also asked about relevant documents which could supplement the analysis. The average interview length was 52 minutes and informed written consent was obtained from participants.

Interview data were supplemented and corroborated with documentary analysis of published and unpublished information, including government policy documents published between 2000 and 2010 [18, 30, 31], reports and strategy documents from interest groups [32, 33], and Department of Health working groups pertaining to diabetes published between 2000 and 2010 [34], meeting agendas, minutes, process evaluation questionnaires, presentations given to and by the EAG, and press releases, media coverage, and parliamentary questions put forward during the lifetime of the EAG process (see Additional file 2 for a table of documents and sources).

### Analysis

All interviews were digitally-recorded and transcribed verbatim by one author (SMH). Data were managed using NVivo 8 software. Thematic content analysis was applied to the data. This approach is considered useful for informing policy development [35]. It allows for the analysis of themes that are anticipated through previous research or knowledge of a theory, and unanticipated themes [36]. First, transcripts were read and open-coded to allow themes to emerge from the data. In the second phase of analysis, the Multiple Streams Theory [21] was used as an analytical framework. The theoretical propositions were used to refine the codes and explore relationships between themes. This is in keeping with suggestions from Walt et al. [37] that explicit attention to a theory does not imply a reductionist approach to analysis but rather provides coherence and potential avenues for linking themes and concepts.

Participant quotes were fully anonymised including the removal of information on position or profession within the health system. Interview data were triangulated with other sources such as information from the documentary analysis. Documents were analysed using a combination of content and thematic analysis [38]. For example, content analysis of media coverage, meeting agendas, and minutes were used to establish timelines and stakeholder involvement in the policy community. Thematic analysis of previous policy documents was used to examine the origins, stability, and progress of priorities and policy proposals. It was also used to explore the coherence between published documents and stakeholder accounts of the aims, role, and remit of the EAG.

## Results

This section presents the interview findings supplemented with information from the documentary analysis. A number of themes emerged, including the perception of diabetes as an obvious problem with nationally and internationally recognised solutions, the unmet expectation of implementation due to internal (lack of authority and dedicated funding) and external barriers (waning support, financial crisis, organisation change), and the importance of timing and opportunities for incremental progress. The themes are organised according to the key features of the Multiple Streams Theory.

### Problem recognition resulting from an accumulation of factors

Diabetes was considered *“a glaring omission”* in the health policy arena in Ireland. The rationale put forward by interviewees as to why diabetes was assigned an EAG was in line with the three streams in the Multiple Streams Theory (Table 1). It was a costly epidemic (problem stream), there was existing evidence and local groundwork to build on (ideas within the policy stream), and health care professionals and non-governmental organisations were *“*lobbying” for change (pressure in the political stream). One of the main approaches to problem definition according to the Multiple Streams Theory is through international comparison and the EAG report itself stated that “*most people knew services in Ireland were behind that (which were) available to their counterparts elsewhere in the developed world*” (p. 2) [39].

**Table 1:** **Rationale for assigning an EAG to diabetes mapped to Kingdon’s streams**

Stream	Quote
**Problem stream**	
Cost	“*Well diabetes is 15*% *of our total health-spend, a major epidemic coming at us, and if* that *can’t hit a policy agenda what can!*” “…*regardless of whether it was being driven by the HSE, health care professionals were lobbying and doing it anyway*.” Hence, there was support within the political stream as people were advocating for change.
Cost and complications	“*It permeates many other medical illnesses. It has a very big impact on cardiac disease and a very big impact on costs and complications of diabetes.*”
Epidemic of growing importance	“*We keep talking about this ticking time bomb but it’s just getting more and more of an issue. And as our population ages it’s becoming more of an issue.*”
Lack of policy in the area	“*It’s well known that in Ireland we have a very poorly organised diabetic care system so I think lots of people in senior positions both in the Dept. of Health and the HSE know that, so it was a glaring omission. We have a cardiovascular strategy and a cancer strategy…*”
**Policy stream**	
Existing groundwork	“…*there were some things that were happening on the ground, I mean regardless of whether it was being driven by the HSE, health care professionals were lobbying and doing it anyway.*”
**Politics stream**	
Interested groups and individuals advocating for change	“*I think as well there were a lot of soldiers’ voices in diabetes and a lot of influence in media at that time so that’s why it was seen as the one most in shape or most ripe for movement on.*”

Although those interviewed felt the reasons for choosing diabetes were obvious, problem recognition at a national level was a gradual process; the result of an accumulation of factors which led to the realisation within the HSE “*that they couldn’t possibly cope with it in the traditional way that they had been*”. The accumulation of factors (Table 1) contributed to the perception of diabetes as “*most ripe for movement*” in terms of reorganising chronic disease management in the health system.

“*I suppose if you look at the wider context and you were to take an example condition where you felt that you could try and pioneer how we could revolutionize how health care is delivered in this country, diabetes is a perfect example.*”

The desire for change and the opportunity for implementation were the most commonly cited reasons among health care professionals for joining the EAG. The expectation of implementation was a strong motivation among participants given their previous experience of *“talking shops”* where reports were drafted and *“lost in the wilderness”*.

“*There was a lot of anxieties going into the EAG because a lot of people who were in that group had been involved in writing documents before that were gathering dust on the shelf. Here was another report and more paper work. And you got that from a lot of your colleagues who weren’t even on the EAG as well.*”

Interviewees thought that the EAG was going to be different *“to what happened before”* as it was internally driven by the HSE and had a remit to “*make recommendations to and be involved with HSE management in implementing them*” according to the EAG report (p. 2) [39].

### Agenda setting and developing proposals in the policy stream

The group followed a rational process through priority setting, analysis of alternatives in subgroups and selection of proposals to put forward to the HSE for approval. The relatively quick formulation of proposals within the group was facilitated by agreement on the main priorities which were considered a ‘no-brainer’. As one interviewee commented “*it stands out a mile what needs to be done. Anyone that’s involved in diabetes care could see what was missing.*” Priorities identified at the first meeting included the need for national retinopathy screening, improvement of podiatry services, a national diabetic register, support for patient education, a national model of care, and agreed guidelines for management.

**Figure 2:**
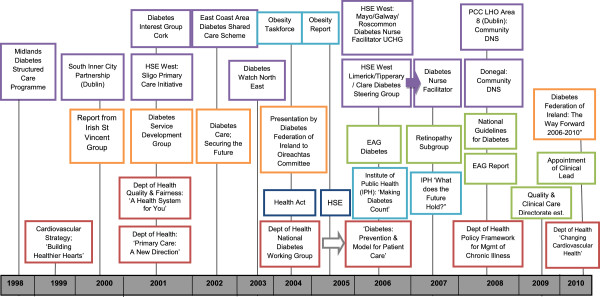
**Overview of policy and practice developments from 1998–2010.**

The proposals considered by the EAG were heavily influenced by previous reports, local loyalties, and existing care arrangements. The reiteration of existing proposals is part of the ‘softening up’ process in the policy stream in which ideas are floated and acceptance is built up over time. The EAG was established against a backdrop of previous attempts to improve the provision of diabetes services. The timeline in Figure 2 illustrates the accumulation and alignment of bottom-up and top-down activities by various groups including professional organisations, academic groups, and diabetes charities. It also illustrates the frequency with which these reports and recommendations were published. In 2000, the Irish St Vincent Group identified priorities including a national retinopathy screening programme, enhanced paediatric services, and better integration between providers. These priorities, and others such as podiatry services and patient education, emerged in several reports over the course of the next decade [32, 34]; by 2008, there were “*3 different processes saying the same thing*”.

Proposals from diabetes-specific groups were aligned with the wider national health strategy; chronic disease management protocols and integrated care arrangements had previously been advocated in the national health strategy [30] and primary care strategy [31] and the chronic disease management framework recommended the development of disease management programmes, proposing the EAG as the starting point [18]. Furthermore, the group was influenced by the strong international consensus on optimal diabetes care.

“*Several times people used the old phrase ‘there’s no point in reinventing the wheel’. Things that are important in Scotland or Denmark are likely to be important in Ireland. So our set of priorities and indeed our standards for diabetes care would have been modelled on the UK equivalent, probably dressed up a little bit in the Irish context …*”

Deciding which priorities to tackle first was largely driven by the likelihood of success. Hence, the need for a national retinopathy screening programme was to the forefront of the agenda; it was not “*the number one priority, it was the number one chance of success*”. Table 2 outlines why retinopathy screening was considered a *“quick win”* proposal, in line with the criteria for survival in the policy stream: technical feasibility, alignment with dominant values, and acceptability in light of future constraints. The feasibility and acceptability of implementing a national screening programme was considered in detail in a dedicated framework document outlining the structure, manpower requirements, governance, and procurement.

**Table 2:** **Factors underpinning retinopathy screening as a viable proposal**

Determinants of viability	Quote
Regional groundwork	“*That* [retinopathy] *came out because there was already movement towards it and because it was something that could be delivered upon. There was already a mobile clinic up and running in the West and was showing great results and great compliance. So it was already on the agenda. And as I said we were practical enough to hitch our wagon to something.*”
International evidence	“…*the fact that the eye screening service had been developed in the North West* [of Ireland]*, that it was based on international evidence and it was very much they had looked to their colleagues and their counterparts in the UK and in Scotland. So it was very much looking beyond Ireland at was is the best way of delivering the screening programme.*”
Financial commitment	“*There was some money for retinopathy*” to extend screening, so it was “*obviously picked because it was already in progress.*”

In contrast, developing recommendations for a national model of care that would be acceptable to different professions and applicable in different regions was described as “*the poisoned chalice*” of the process. Participants referred to a historical “*tug of war*” between primary and secondary care over the management of diabetes and competition for limited resources, which led to “*entrenched positions*” on the ideal care arrangement. The development of proposals for an integrated model of care illustrated the process of diffusion and acceptance within the policy stream as participants referred to the gradual realization that “*it really was impossible for either service to look after Type 2 diabetes alone*”.

Given the different ways services were organised around Ireland, “*there was this kind of fudge that we needed to organise*” so that the model could take account of local capacity and acceptability. In keeping with the concept of repackaged policy ideas, the established models of shared and structured care were repackaged as ‘integrated care’. The evolution of recommendations represented the emergence of consensus rather than complete agreement as some participants expressed doubt about the technical feasibility of implementing the model of care.

### Attempting to couple the problem and policy streams

Participants referred to a number of strategies used to increase the chance that proposals would be accepted and implemented by the HSE. In the first instance, the EAG was cognisant of the need to align proposals with national policy and “*not (to) wander off message.*” Secondly, the group tried to contextualize proposals and fit with the future direction of the HSE, seizing opportunities to address individual aspects of their overall plan. For example, early in the process the group was asked to prepare submissions to put forward to the HSE to inform its budget allocation process. The group prioritised community diabetes nurse specialist positions to act as a link with primary and secondary care services. According to one interviewee, the group agreed “*that if there was one thing we could get to happen it would be community diabetes nurses and my recollection of the submission was prioritizing that link to primary care teams and primary care networks…that was clearly the direction of travel at the HSE at that time.*” Finally, the group recommended the establishment of regional Diabetes Services Implementation Groups, as proposed in previous reports [32, 34], to increase the feasibility of implementing the recommendations at local level. This proposal, which had limited cost implications, is now directly linked into the national Clinical Care Programme for Diabetes. It was described by one interviewee as one of “*the biggest outputs of the EAG*”.

### Getting onto the decision agenda

The recommendations were finalised by the EAG in September 2007. This was followed by a series of meetings between members of the EAG and “*the* [CEO’s] *kitchen cabinet*”. During the approval process, the costing framework and gap analysis requested by senior management “*frightened off*” interest and generated a “*fear of creating need*”*,* according to interviewees. Enthusiasm and commitment had waned at higher levels of the HSE, and the EAG had become “*a headache*”. Senior HSE managers insisted that recommendations should be cost neutral and new projects would need to come from existing resources. One participant reflected on the inevitability “*that this is going to cost money and more importantly it is going to cost new posts because the fact of life is that diabetes is very poorly served in Ireland.*” Endorsement of the EAG proposals came in September 2008 at a meeting with senior management and the CEO; this was perceived as the final hurdle to implementation. However, there was a continuing sense of uncertainty within the group: “*was that an endorsement or was that not an endorsement; was that policy or not policy? It wasn’t clear…*” The lack of clarity extended to which person or persons made decisions in the health system in contrast to clinical decision-making:

“*You will never get the name of the person who is making the decision, never! And if they do, they’ll have made a decision and changed to another department…* [It] *is very odd to our brains as clinicians because every single day, all day every day, we’re making finite life-changing decisions.*”

Our analysis echoed Kingdon’s distinction between the governmental agenda (the list of subjects getting attention) and the decision agenda (the list of subjects considered for active decision-making). According to one participant “*It’s one thing signing of the policy document and there are a lot of policy documents, it’s another thing to see it into action*”*.* Getting to the management table was not the final step before action; diabetes joined a list of issues for prioritisation by the executive. While hindsight imposed a sequence on the approval process, at the time the path to implementation was not well-defined. The gap between approval and action was one of the most commonly cited frustrations of the EAG experience.

### Lack of support in the politics stream

According to interviewees, the reasons for the implementation gap were the lack of authority within the group, and a lack support for the recommendations outside the group. Firstly, there was a perceived lack of “*authority and the mandate to go off and make things happen*” in the group. The group “*was given a brief but it wasn’t given all the powers. It was purely advisory and it wasn’t very clear how the advisory function would be translated to implementation*”. There was no specific budget to make changes or allocate resources, and there was an absence of representation within the group to drive change from inside the system.

Earlier changes to the structure of the health system meant the Department of Health was responsible for policy making while the HSE was charged with service delivery. Hence, the EAG was left in an “*invidious position*” without “*the power to make policy or the power to implement it*”. Secondly, interviewees described the “*non-movement, non-commitment and non-support*” for implementation from the central executive. According to one participant, two conditions necessary for implementation were that “*the executive want it to be done and feel required to implement what is proposed*”. The failure to link the formulation stage with implementation created a situation whereby “*the recommendations aren’t owned by those that might be able to implement them*”. The lack of impetus from within the HSE, a deficiency in the political stream, corresponds to Kingdon’s observation that sometimes a cause lacks sufficiently powerful supporters leading to inertia.

### Closing of the policy window

The EAG process reflected the dynamic and unstable environment in which policy is formulated and a number of interviewees reflected on the unfortunate timing of the recommendations. There were two changes which led to the closing of the policy window: the increasing economic recession and the establishment of the Quality and Clinical Care Directorate within the HSE. The EAG was seen as an “*overdue opportunity to bring services for all people with diabetes in Ireland into the 21st Century*” (p. 2) [39]. However, by the time the recommendations were formulated, health service budgets were contracting and there was a recruitment embargo in place in the health system. One interviewee admitted “*it was more likely that more of it could have been implemented a few years ago because we were probably a little bit more financially secure.*”

During the process, the establishment of the Clinical Care Directorate and its disease or sector-specific Clinical Care Programmes was identified as a policy window. The proposal to establish a clinical care programme for diabetes with a dedicated national lead was brokered by a number of policy entrepreneurs. One interviewee referred to the instrumental role of the Diabetes Federation of Ireland, which had ‘claim to a hearing’ with the Minister for Health, as described in the Multiple Streams theory. Acceptance at the highest levels of governance in the health system was crucial as the group “*didn’t really get an impetus for the national lead until we actually got* [the Minister for Health] *on board*”. Considering the other criteria for survival in the policy stream, the role of a national clinical lead had already been successfully established for cancer care (technical feasibility); hence, the proposal was in line with values in the health system at that time (acceptability). It was also mutually beneficial as it kept diabetes on the national agenda and introduced national leadership. It was also a potential “*quick win*” for the newly established Clinical Care Directorate; due to the groundwork of the EAG they “*didn’t have to start from scratch*” formulating recommendations.

The establishment of a Clinical Care Programme for Diabetes revised the expected trajectory of the EAG. Within the politics stream, these changes in jurisdiction altered positions of power and shifted priorities on the agenda. The expressed desire among EAG members for involvement in implementation was superseded by the new structure which was to be “*a completely separate programme with a* [new] *lead and they would do it their way.*” There is some overlap in membership between the groups and most participants felt there was commitment to the recommendations “*in a general way*”, leaving room for discretion about the order of and extent to which proposals were implemented. At the time of interview (late 2010), there was ambiguity and scepticism about the fate of the EAG’s recommendations in light of organisational changes. However, a number of participants suggested that the Clinical Care Programme had more chance of success as it had been afforded a greater opportunity to bridge the implementation gap.

“*The implementation piece was never tied in…Its changing now with the care groups being set up and we now have leads for diabetes and its tied into the executive and that’s where the EAG was weak.*”

## Discussion

The aim of this study was to examine the development of recommendations by the EAG for diabetes as an instrumental case study of the health policy formulation process. This paper applied Kingdon’s Multiple Streams Theory to understand both the content and the context of the recommendations. Our analysis suggests that the problem stream and policy stream were on course to converge, as a result of agreement on the growing problem of diabetes and accumulating national and international evidence on how best to manage the condition. The EAG assumed the politics stream was also on course to converge with the other streams, having been established by the HSE, which had the remit for policy implementation. Many stakeholders also envisaged having a role in implementation following approval of the recommendations. However, in the politics stream, there was waning support from health service management, changes to the organisational structure, and seriously reduced financial capacity in the health system. According to Kingdon, early optimism and awareness of the need for change is often replaced by the realisation of the costs of action [21]. Thus, the politics stream in effect dried up and the policy window remained closed.

While alternative theories of the policy process were considered during analysis [40, 41], the Multiple Streams Theory provided the best explanatory fit with the data [21]. The rational model of policy making did apply to decision making at a micro-level within the EAG group itself as the group followed a logical sequence: priority setting, evidence appraisal, analysis of alternative options in subgroups, and consensus on proposals to put forward to the HSE for approval. However, it fails to take into account external influences such as the barriers and opportunities presented within the wider health system. Walt and Gilson argue that the policy process occurs within particular contexts with which it interacts [28]. The EAG and its recommendations were embedded within a health system that was in a state of flux. While certain existing commitments in other priority areas were being met, for example the development of symptomatic breast services, there was little new investment of resources in the health system at the time. The emphasis was on cost saving, reducing staff numbers, and doing more with less [23]. The EAG process was ultimately superseded by these priorities.

The Multiple Streams Theory of the policy process was developed in the United States in 1990s, yet it remains a popular choice for understanding the health policy process in Europe [42–44]. Previous studies identified the lack of problem definition and the absence of concrete proposals as barriers to the policy window [44, 45]. However, in this study, there was consensus on what should be done and how to do it but the political climate was unfavourable. Our analysis indicated overall agreement on diabetes as a health system priority and consensus on the best approach to its management influenced by national and international experience. Kingdon’s concept of a ‘softening up’ process, whereby ideas are reiterated and repackaged over time, fits with the EAG process, which took place against a backdrop of numerous proposals and reports on how to improve services. This influence is often referred to as path dependency, whereby decision-making is constrained by previous proposals and historical context [46].

International approaches to diabetes care were considered in light of the Irish context and local circumstances. This is similar to the findings of Tervonen et al. [42], who examined the transfer of the WHO *Health for All* policy. They found that as Portugal and Finland lagged behind in terms of public health policy in Europe, the policy stream was more open to policy transfer but only for elements of the policy that fit with the policy context of that country. Although Ireland could be viewed as lagging behind internationally in terms of the organisation and integration of diabetes care, the EAG were keen to contextualise proposals to make implementation more feasible.

The findings of this study highlight the dynamic unstable nature of the policy process and the fleeting opportunity for change. The convergence of streams is not considered a passive or automatic process within the Multiple Streams Theory [21]; policy entrepreneurs look for opportunities to couple proposals and problems. There were a number of examples of the group seizing opportunities to make incremental progress on individual elements of their plan such as requesting funding for diabetes nurse specialist posts which have now been funded as part of the National Clinical Care Programme and efforts involving members of the EAG to introduce a national clinical lead for diabetes in Ireland. Policy entrepreneurs are often driven by desire for change and the opportunity for implementation, referred to by a number of our participants as the main reason for joining the EAG. Exworthy et al. [47] advise that local expectations are often dashed by the central policy approach and vice versa, hence there is a need to align top-down and bottom-up expectations regarding implementation. In this study, there were unmet expectations regarding implementation and the group’s role in that process. There is a risk of policy fatigue or burnout among stakeholders who regularly engage in policy formulation but see little return on their investment in terms of demonstrable change.

### Strengths and limitations

We were unable to recruit all members of the EAG to take part in the study and those who did not participate may have had unique and valuable insights into the process. However, a consistent and rich case description was built through in-depth interviews supplemented with the analysis of internal and external documents. Interviews took place almost two years after the EAG process and we acknowledge that participants’ accounts were inevitably shaped by their position at the time of interview and changes subsequent to the EAG process. However, the timing of the study allowed us to discuss the fate of the EAG proposals and explore participants’ attitudes to the newly established National Clinical Care Programme. This programme is still in its infancy but incremental progress has been made in certain priority areas, including the introduction of a national retinopathy screening programme in 2013.

Studies of the policy process are often criticised for providing a description rather than an explanation of what happened [48], and for the absence of theory to underpin analysis [37]. This is one of the first studies to examine the policy process within the Irish health system and contributes to both the theoretical and practical principles of policy formulation. This study goes beyond description to explanation, illustrating how external events, such as an economic crisis, suppressed internal support from senior management, leaving the EAG powerless to implement change. Although specific to diabetes, the study captured a number of commonly identified features of the policy-making process, including bargaining or ‘horse-trading’, and covert decision making [28, 49]. In terms of practice, a number of policy tactics were identified which could increase the chances of acceptance and approval of policy proposals within the health system. Retrospective analysis of success and failure in policy making creates a learning environment for those involved in the policy process [50].

## Conclusions

The Multiple Streams Theory proposes a more fluid iterative approach to policy making which reflects the dynamic nature of the diabetes policy process in Ireland and the effects of unexpected shocks to the system. The results highlight the limitation of rational policy making in the face of organisational and fiscal upheaval. The outcome of the EAG process could be viewed as incremental progress towards implementation as a number of EAG proposals continue to evolve as part of the National Clinical Care Programme for Diabetes. This may represent the opening of a new policy window for diabetes services in Ireland, the challenge will be maintaining momentum and interest in the absence of dedicated resources.

## Authors’ information

Dr Sheena Mc Hugh (PhD), Post-doctoral Research Fellow, Department of Epidemiology & Public Health, University College Cork, Cork, Ireland. Prof. Ivan J. Perry (MD, PhD), Professor and Head of Department of Epidemiology & Public Health, University College Cork, Cork, Ireland. Prof. Colin Bradley (MB, BCh, BAO, MD), General Practitioner, Professor and Head of Department of General Practice, University College Cork, Cork, Ireland. Prof. Ruairí Brugha (MB, BCh, BAO, MD), Professor and Head of the Department of Epidemiology and Public Health Medicine, Royal College of Surgeons Ireland, Dublin, Ireland.

## Electronic supplementary material

Additional file 1: Table S1: Topic guide used for semi-structured interviews. (PDF 418 KB)

Additional file 2: Table S2: Secondary sources of information included in documentary analysis. (PDF 221 KB)

## Abbreviations

EAG: Expert Advisory Group
HSE: Health Service Executive.

## References

[1] International Diabetes Federation-Europe, Federation of European Nurses in Diabetes (2005). Diabetes – The Policy Puzzle: Towards Benchmarking in the EU 25.

[2] Department of Health and Children (2006). A Strategy for Cancer Control in Ireland.

[3] Wagner EH, Austin BT, Davis C, Hindmarsh M, Schaefer J, Bonomi A (2001). Improving chronic illness care: translating evidence into action. Health Aff.

[4] Wild S, Roglic G, Green A, Sicree R, King H (2004). Global prevalence of diabetes: estimates for the year 2000 and projections for 2030. Diabetes Care.

[5] Jiang HJ, Stryer D, Friedman B, Andrews R (2003). Multiple hospitalizations for patients with diabetes. Diabetes Care.

[6] Zhang P, Zhang X, Brown J, Vistisen D, Sicree R, Shaw J, Nichols G (2010). Global healthcare expenditure on diabetes for 2010 and 2030. Diabetes Res Clin Pract.

[7] Diabetes Care and Research in Europe (1990). The Saint Vincent Declaration. Diabet Med.

[8] Firth R (2000). Existing facilities for diabetes care in the Republic of Ireland and developments required. Ir J Med Sci.

[9] Griffin S (2001). The management of diabetes. Br Med J.

[10] Maier M, Knopp A, Pusarnig S, Rurik I, Orozco-Beltran D, Yaman H, van Eygen L (2008). Diabetes in Europe: role and contribution of primary care position paper of the European Forum for Primary Care. Qual Prim Care.

[11] Mc Hugh S, O’Keeffe J, Fitzpatrick A, de Siún A, O’Mullane M, Perry I, Bradley C (2009). Diabetes care in Ireland: a survey of general practitioners. Prim Care Diabetes.

[12] O'Donnell M, de Siún A, O'Mullane M, Smith D, Bradley C, Finucane F, Dinneen S (2013). Differences in the structure of outpatient diabetes care between endocrinologist-led and general physician-led services. BMC Health Serv Res.

[13] Smith S, Bury G, O’Leary M, Shannon W, Tynan A, Staines A, Thompson C (2004). The North Dublin randomized controlled trial of structured diabetes shared care. Fam Pract.

[14] Brennan C, Harkins V, Perry I (2008). Management of diabetes in primary care: a structured-care approach. Eur J Gen Pract.

[15] Mc Hugh S, Marsden P, Brennan C, Murphy K, Croarkin C, Moran J, Harkins V, Perry IJ (2011). Counting on commitment; the quality of primary care-led diabetes management in a system with minimal incentives. BMC Health Serv Res.

[16] International Diabetes Federation-Europe, Federation of European Nurses in Diabetes (2008). Diabetes – The Policy Puzzle: Is Europe Making Progress?.

[17] Health Service Executive (2006). Expert Advisory Groups; An Introduction.

[18] Department of Health & Children (2008). Tackling Chronic Disease. A Policy Framework for the Management of Chronic Disease.

[19] Expert Advisory Group National Retinopathy Screening Committee (2008). Framework for the Development of a Diabetic Retinopathy Screening Programme for Ireland.

[20] Harkins V (2008). A Practical Guide to Integrated Type 2 Diabetes Care.

[21] Kingdon J, Thurber J (2003). Agendas, Alternatives, and Public Policies.

[22] Keegan C, Thomas S, Normand C, Portela C (2013). Measuring recession severity and its impact on healthcare expenditure. Int J Health Care Finance Econ.

[23] Thomas S, Burke S, Barry S (2014). The Irish health-care system and austerity: sharing the pain. Lancet.

[24] Health Service Executive (2013). Health Service National Performance Assurance Report.

[25] Creswell JW (2007). Qualitative Inquiry & Research design: Choosing among 5 Approaches.

[26] Yin RK (2009). Case Study Research. Design and Methods.

[27] Dobrow MJ, Goel V, Lemieux-Charles L, Black NA (2006). The impact of context on evidence utilization: a framework for expert groups developing health policy recommendations. Soc Sci Med.

[28] Walt G, Gilson L (1994). Reforming the health sector in developing countries: the central role of policy analysis. Health Policy Plan.

[29] Bowen S, Zwi A (2005). Pathways to “evidence-informed” policy and practice: a framework for action. PLoS Med.

[30] Department of Health & Children (2001). Quality and Fairness ‘A Health System for You’.

[31] Department of Health & Children (2001). Primary Care – A New Direction.

[32] Clarke A (2002). Diabetes Care: Securing the Future. Report of the Diabetes Service Development Group.

[33] Diabetes Federation of Ireland (2006). The Way Forward 2006 – 2010; Strategy of the Diabetes Federation of Ireland.

[34] Department of Health & Children (2006). Diabetes: Prevention & Model for Patient Care.

[35] Braun V, Clarke V (2006). Using thematic analysis in psychology. Qual Res Psychol.

[36] Mays N, Pope C (2006). Qualitative Research in Health Care.

[37] Walt G, Shiffman J, Schneider H, Murray S, Brugha R, Gilson L (2008). ‘Doing’ health policy analysis: methodological and conceptual reflections and challenges. Health Policy Plan.

[38] Bowen GA (2009). Document analysis as a qualitative research method. Qual Res J.

[39] Expert Advisory Group for Diabetes (2008). Diabetes Expert Advisory Group First Report: April 2008.

[40] Simon H (1979). Rational decision making in business organizations. Am Econ Rev.

[41] Sabatier PA, Weible CM, Sabatier PA (2007). The advocacy coalition framework: Innovations and clarifications. Theories of the Policy Process.

[42] Tervonen-Gonçalves L, Lehto J (2004). Transfer of health for all policy–what, how and in which direction? A two-case study. Health Res Policy Syst.

[43] Caldwell SE, Mays N (2012). Studying policy implementation using a macro, meso and micro frame analysis: the case of the Collaboration for Leadership in Applied Health Research & Care (CLAHRC) programme nationally and in North West London. Health Res Policy Syst.

[44] Tynkkynen L-K, Lehto J (2009). An analysis of ophthalmology services in Finland-has the time come for a public-private partnership?. Health Res Policy Syst.

[45] Walhart T (2013). The application of Kingdon’s Multiple Streams Theory for human papillomavirus-related anal intraepithelial neoplasia. J Adv Nurs.

[46] Wilsford D (1994). Path dependency, or why history makes it difficult but not impossible to reform health care systems in a big way. J Public Policy.

[47] Exworthy M, Berney L, Powell M (2002). ‘How great expectations in Westminster may be dashed locally’: the local implementation of national policy on health inequalities. Policy Politics.

[48] Gilson L, Raphaely N (2008). The terrain of health policy analysis in low and middle income countries: a review of published literature 1994-2007. Health Policy Plan.

[49] Exworthy M (2008). Policy to tackle the social determinants of health: using conceptual models to understand the policy process. Health Policy Plan.

[50] McAulliffe E, McKenzie K (2007). The Politics of Health Care: Achieving Real Reform.

