# Supplementary material for: Developing recommendations to improve the quality of diabetes care in Ireland: a policy analysis
Source: Health Res Policy Syst. 2014 Sep 18;12:53. doi: 10.1186/1478-4505-12-53 (PMC4177249; doi:10.1186/1478-4505-12-53)
Supplement: Supplementary file 1 — Additional file 1: Table S1: Topic guide used for semi-structured interviews. (PDF 418 KB) [file 12961_2014_350_MOESM1_ESM.pdf]

Table S1 Topic guide used for semi-structured interviews

**PARTICIPANT & EAG**	Your position now & then<br>How did you get involved<br>Who decided EAGs & why?<br>What led up to EAGs? Why now?<br>Why diabetes?<br>What were there particular challenges in diabetes care at the time?
**MEMEBERSHIP**	Other members selected<br>Remit for group<br>Your role within group (did it change)<br>Your objectives/priorities<br>Your expectations for the group
**DEVELOPING POLICY**	Objectives from the outset…<br>How were priorities identified<br>- who brought them to table<br>What issues were to the forefront, most occupied with?<br>- How recommendations developed
**ALTERNATIVES**	Alternative solutions discussed<br>- sharing ideas<br>How were alternatives evaluated<br>Why disregard them<br>Topics of confusion or disagreement<br>How were differences of opinion handled? (consensus)<br>Was there bargaining involved? In what way…
**CONTENT + EVIDENCE**	Existing/ available evidence<br>Efforts to produce/gather evidence<br>Types of evidence drawn on: expertise, international, research<br>How did presentations inform report? Was information used?
**SUB-GROUP**	Why that subgroup/topic<br>(Integrated care, how was that model developed?)<br>Bringing ideas to the larger group for discussion/agreement?
**FINALISING REPORT**	Any crisis points in the process?<br>How did the approval process work with the HSE?<br>- Timeline, stakeholders, decisions, progress, outcomes
**RELATIONSHIPS**	Group dynamic- influence, leadership, partnership, networks<br>Resistance, enabling factors<br>People resisting/enabling<br>Other Barriers & Facilitators

**CONTEXT**	Key milestones/crises in diabetes<br>Previous efforts – was this process different, advantages/disadvantages of this attempt<br>Influence of other policies, changes in HSE…<br>Financial implications/ Economic situation<br>Current resources: staff, skills, organization.<br>Media Role? Role of advocacy groups?
**IMPLEMENTATION**	What was the expectation once recommendations were agreed?<br>Reaction of diabetes community: widely accepted?<br>Critical factors for implementation<br><br>Should the content differ?<br>What could have been done differently<br>Balance of goals & priorities<br>Use of EAGs for policy making
