# Supplementary material for: Developing recommendations to improve the quality of diabetes care in Ireland: a policy analysis
Source: Health Res Policy Syst. 2014 Sep 18;12:53. doi: 10.1186/1478-4505-12-53 (PMC4177249; doi:10.1186/1478-4505-12-53)
Supplement: Supplementary file 2 — Additional file 2: Table S2: Secondary sources of information included in documentary analysis. (PDF 221 KB) [file 12961_2014_350_MOESM2_ESM.pdf]

## Additional file 2:

### Table S2 Secondary sources of information included in documentary analysis

| Type | Source |
|---|---|
| **National-level Policy 2000-2010** | |
| National Health Strategy 2001: *"Quality & Fairness; a Healthy System for you "* **(279)**. | Department of Health and Children (DOHC) 2001 |
| Primary Care Strategy 2001: *"Primary Care: A New Direction"* **(182)** | DOHC 2001 |
| *"Tackling Chronic Disease: A policy framework for the management of Chronic Disease."* (2008) **(11)** | DOHC 2008 |
| **Position Statements on Diabetes 2000-2010** | |
| *"Diabetes: Prevention & Model for Patient Care"* (2006). | Diabetes Working Group established by DOHC in 2004 |
| *"Diabetes Care; Securing the Future" (2002)* | Diabetes Service Development Group linked to Diabetes Federation of Ireland (DFI) |
| *"Diabetes Federation of Ireland: The Way Forward 2006-2010"* | DFI Strategy Paper 2010 |
| **EAG Process Documentation 2006-2010** | |
| Meeting Agendas | Online, health service administration, group members |
| Meeting Minutes | |
| Evaluation questionnaires of the process | |
| Copy of some presentations to the group | |
| Official press release from HSE | |
| **Media 2006-2010** | |
| HSE Press Releases | Online |
| Coverage in national newspapers, online health forums | |
| **Other 2006-2010** | |
| Parliamentary Questions | Online |
| Oireachtas Reports | |
